# Novel avian paramyxovirus (APMV-15) isolated from a migratory bird in South America

**DOI:** 10.1371/journal.pone.0177214

**Published:** 2017-05-09

**Authors:** Luciano Matsumiya Thomazelli, Jansen de Araújo, Thomas Fabrizio, David Walker, Dilmara Reischak, Tatiana Ometto, Carla Meneguin Barbosa, Maria Virginia Petry, Richard J. Webby, Edison Luiz Durigon

**Affiliations:** 1 Laboratorio de Virologia Clinica e Molecular do Instituto de Ciencias Biomedicas, Universidade de Sao Paulo, Sao Paulo, Brazil; 2 Department of Infectious Diseases, St. Jude Children’s Research Hospital, Memphis, Tennessee, United States of America; 3 Laboratório Nacional Agropecuário, Campinas, Sao Paulo, Brazil; 4 Universidade do Vale do Rio dos Sinos, Sao Leopoldo, Rio Grande do Sul, Brazil; SWEDEN

## Abstract

A novel avian paramyxovirus (APMV) isolated from a migratory bird cloacal swab obtained during active surveillance in April 2012 in the Lagoa do Peixe National Park, Rio Grande do Sul state, South of Brazil was biologically and genetically characterized. The nucleotide sequence of the full viral genome was completed using a next-generation sequencing approach. The genome was 14,952 nucleotides (nt) long, with six genes (3’-NP-P-M-F-HN-L-5’) encoding 7 different proteins, typical of APMV. The fusion (F) protein gene of isolate RS-1177 contained 1,707 nucleotides in a single open reading frame encoding a protein of 569 amino acids. The F protein cleavage site contained two basic amino acids (VPKER↓L), typical of avirulent strains. Phylogenetic analysis of the whole genome indicated that the virus is related to APMV-10, -2 and -8, with 60.1% nucleotide sequence identity to the closest APMV-10 virus, 58.7% and 58.5% identity to the closest APMV-8 and APMV-2 genome, respectively, and less than 52% identity to representatives of the other APMVs groups. Such distances are comparable to the distances observed among other previously identified APMVs serotypes. These results suggest that unclassified/calidris_fuscicollis/Brazil/RS-1177/2012 is the prototype strain of a new APMV serotype, APMV-15.

## Introduction

Avian paramyxovirus (APMV) belongs to the genus *Avulavirus* of the family Paramyxoviridae. Members of this family are characterized by pleomorphic enveloped particles that contain a single-stranded, negative sense RNA genome of 13 to 19 kb [1,2]. APMV is classified into twelve distinct serotypes (APMV-1 to -12) by ICTV Virus Taxonomy 2016, based on serological tests, such as hemagglutination inhibition (HI) or neuraminidase inhibition (NI) tests and confirmed by phylogenetic analysis [3]. In August 2016, three APMVs isolated from a rockhopper penguin in the Falkland Islands in 2007, a common snipe in France in 2010 and an Eurasian wigeon in Italy 2005 were designated as new serotypes, APMV-10, -11 and -12, respectively [4,1,2,3]. Two unclassified *Avulavirus* isolated from wild birds in Japan in 2000 and 2011 have been proposed to be additional serotypes, APMV-13 and APMV-14 respectively [5,6,7,8]. Here, based on phylogenetic analysis, serological and biological tests, we propose a new serotype, APMV-15. Since 2005, the Laboratory of Virology of Institute Biomedical Science—USP has conducted an active surveillance programme in Brazil aimed at detecting the presence of avian influenza virus (AIV) and Newcastle disease viruses (NDV) in wild birds to assess the risk of introduction of such viruses to poultry. In this surveillance, AIV and NDV have been isolated from North American migratory birds wintering in Brazil [9,10]. As part of this programme, tracheal/cloacal swabs were taken from a white-rumped sandpiper (*Calidris fuscicollis*, order Charadriiformes), captured in April 2012 in the Lagoa do Peixe National Park, Rio Grande do Su state, in southern Brazil, from which a hemgglutinating agent was isolated in embryonating chicken eggs.

## Methods and results

All procedures involving wild birds were approved by the Brazilian Society of Laboratory Animal Science (SBCAL) of the University of Sao Paulo (Permit Number: 105, page 74, book 2) and licensed by the Ministerio do Meio Ambiente-MMA at the Instituto Chico Mendes de Conservacao da Biodiversidade (ICMBio/SISBIO), Number 25895–1.

The tracheal/cloacal swab sample, RS-1177, collected and processed as previously described [10] was positive in Real-time RT-PCR (rRT-PCR) for Influenza A virus; subsequent sequencing directly from the clinical material confirmed the finding (under publication). Nine-day-old embryonating specific-pathogen-free chickens’ eggs were inoculated with swab sample and incubated for 48 hours. Allantoic fluid was harvested from infected eggs and RNA was extracted using the Qiagen RNeasy mini kit (Qiagen, Germantown, MD, USA). The allantoic fluid contained an agent that hemagglutinated chicken erythrocytes but the fluid was negative for influenza A by rRT-PCR suggesting another agent had been isolated. In order to obtain the complete genomic sequence of the hemagglutinating agent (isolate RS-1177), cDNA was generated using a modified version of sequence independent single primer amplification as previously described [11,12]. Briefly, the RNA was reverse transcribed using a tagged random and tagged Poly-A primers with Superscript III (Invitrogen, Carlsbad, CA, USA). The second strand was generated using 3’-5’ exo-Klenow fragment (New England Biolabs, Ipswich, MA, EUA). The double stranded cDNA was then amplified using primers to the tag sequence and Phusion polymerase (ThermoFisher, Waltham, MA, USA). Illumina libraries were prepared from the PCR amplicons using the Nextera XT DNA Library Preparation Kit (Illumina) and run on the Illumina MiSeq at the Hartwell Center for Bioinformatics and Biotechnology at St. Jude Children’s Research Hospital (Memphis, TN, USA). The raw sequence data was processed and analyzed using CLC Genomics Workbench v9.0.1 as follows. The reads were trimmed to remove low quality reads followed by merging overlapping paired reads to generate longer segments. All reads were run in a *de novo* assembly pipeline and generated three contigs with the longest contig being 14,952 nucleotides (nt) with an average coverage of over 9500. A search for open reading frames (ORFs) with a minimum length of 100 amino acids resulted in 12 ORFs identified. The ORFs were blasted and came back as most similar to APMV-2 and APMV-8. A genome organized in a manner consistent with an APMV was identified based on the genomes of APMV-2/Chicken/England/7702/06 and APMV-8/Goose/Delaware/1053/76. The sequence was deposited in GenBank database (Accession number: KX932454).

To confirm the next-generation sequencing we used a conventional hemi-nested RT-PCR previously described to detect the Paramyxoviridae family [13]. The amplicons of polymerase pol gene of 530 bp were sequenced using a Big-Dye Terminator v3.1 Cycle Sequencing Kit (ThermoFisher, Waltham, MA, USA) and sequenced using an ABI PRISM 3130 Genetic Analyzer (Applied Biosystems, Foster City, CA, USA). The partial sequence of Sanger sequencing method matched 100% with next-generation sequencing (GenBank accession number: KY499582).

The complete genome was 14,952 nucleotides (nt) long with a GC content of 41%. The genome organization of the virus was typical of APMV, with six genes (3’-NP-P-M-F-HN-L-5’) encoding 7 different proteins [14]. Theoretical amino acid (aa) lengths of the seven putative proteins were as follows: NP, 464 aa; P and V, 434 aa and 113 aa, respectively; M, 398 aa; F, 569 aa; HN, 604 aa; and L, 2243 aa. Each of six genes of isolate RS-1177 were translated in BioEdit Sequence Alignment Editor software version 7.2.0 [15] and compared by protein-protein BLAST (BLASTp) search with Basic Local Alignment Search Tool on The National Center for Biotechnology Information Homepage (NCBI).

The highest deduced amino acid identities of RS-1177 obtained on Blastp were nucleoprotein (71%) with APMV-10/penguin/Falkland Islands/324/2007; phosphoprotein (39%) APMV-2/Chicken/England/7702/06; matrix protein (52%) APMV-10/penguin/Falkland Islands/324/2007; fusion protein (60%) APMV-8/Goose/Delaware/1053/76; hemagglutinin-neuraminidase (48%) APMV-8/Goose/Delaware/1053/76 and polymerase (60%) with APMV-10/penguin/Falkland Islands/324/2007.

The fusion protein gene of isolate RS-1177 contained 1,707 nt in a single open reading frame encoding a protein of 569 aa. The putative cleavage site of F protein contained a pair of single basic amino acid (VPKER↓L), typical of avirulent AMPV strains.

The complete genome nucleotide sequence of RS-1177 was aligned with representative viruses of the family Paramyxoviridae including sequences of each APMV serotype (APMV-1 to -12) and all unclassified *Avulavirus* (including APMV-13 and -14) available in GenBank (NCBI). For the construction of the phylogenetic trees, the evolutionary history was inferred using the maximum-likelihood method based on the Tamura-Nei model [16] and the Substitution Type “Nucleotide” using MEGA 6 [17].

**Fig 1:**
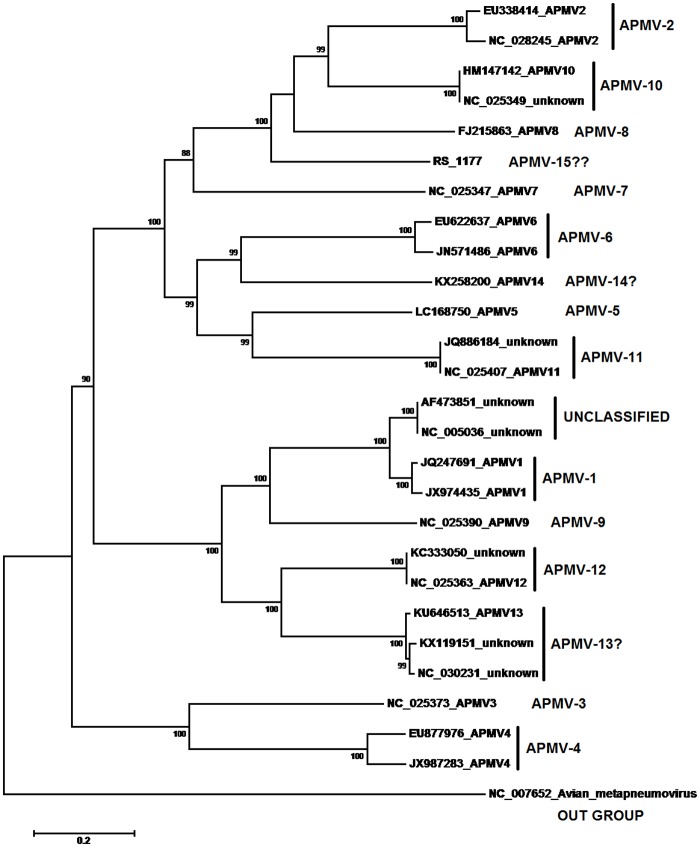
Phylogenetic tree of representative members of *Avulavirus*. Phylogenetic analysis using the Neighbor-joining method with 1,000 bootstrap replicates based on complete genome sequences of APMV-1 to APMV-14 and other unclassified *Avulavirus*. Evolutionary distances were computed using the Kimura 2-parameter substitution model. The scale bar is shown on the bottom left. The numbers at the nodes represent bootstrap values higher than 70. Sequences used were available in public databases and the accession numbers along with their branch data are shown. Label:?- Species not recognized by ICTV;??- New isolate RS-1177.

Phylogenetic analysis of the whole genome of RS-1177 confirmed the closest related to APMVs and indicated that it was closest to APMV-10, -8 and -2, with 60.1%, 58.7% and 58.5% nucleotide identity, respectively. RS-1177 shared less than 52% identity to representatives of the other APMVs groups (Fig 1). For comparison, the identities seen between the closest APMV-12 and APMV-13 are <64%, between APMV-1 and APMV-9 are <61%, and between APMV-2 and APMV-10 are <61.7%. The lowest similarity between APMVs was 43.4% (APMV-1 with APMV-2 and -3); the lowest similarity of isolate RS-1177 was 43.7% with APMV-4 (S1 Table). We consider that the large genetic distance between RS-1177 and other known APMV suggests that it should be considered the prototype strain of a new APMV group, APMV-15, with the full name APMV-15/*calidris_fuscicollis*/Brazil/RS-1177/2012.

The serological characterization and biological tests were conducted in the OIE reference laboratory for avian influenza and Newcastle disease, National Agriculture Laboratory in São Paulo (LANAGRO-SP), according to the World Organization for Animal Health guidelines [18]. In HI tests, antisera against influenza H-1 to H-16 and APMV-1 to APMV-9 (except APMV-5) were used as references. Antisera against APMV-10 to APMV-14 were not available. The following serotypes were used: A/PR/8/34(H1), A/Singapore/1/57(H2), A/Duck/Ukraine/1/63/H3, A/Duck/Shantou/461/2000(H4), A/Tern/South Africa/61(H5), A/Turkey/MA/65(H6), A/FPV/Rostock(H7), A/Turkey/Ontario/6118/68(H8), A/Chicken/Hong Kong/G9/97(H9), A/Chicken/Germany/N/49(H10), A/Duck/England/56(H11), A/Duck/Alberta/60/76(H12), A/Gull/Maryland/704/77(H13), A/Mallard/Astrakhan/263/82(H14), A/Shearwater/Australia/2576/79(H15), A/Shorebird/DE/172/2006(H16), APMV-1/NDV/KS, APMV-2/HOBIN/HIDDENSEE/19/75, APMV-3/Tky/England/1087/82, APMV-4/DUCK/HK/D3/75, APMV-6/DUCK/HONG KONG, APMV-7/Dove/Tn/4/75, APMV-8//GOOSE/DEL/1053/76, APMV-9/Dom./Duck/NY/22/78. In HI tests, RS-1177 showed no cross-reaction with influenza A virus and little or no HI activity against other APMV serotypes. APMV-8 and -2 antisera showed activity against RS-1177 (1:160). All other antisera showed activity ≤40, considered negative (S2 Table).

To determine virulence of isolate RS-1177, intracerebral pathogenicity index (ICPI) test was performed using ten one-day-old SPF white leghorn chicks as previously described [18]. Briefly, 10-fold dilutions of fresh infective allantoic fluid (>16HAU) was injected intracerebrally. The birds were examined every 24 hours for 8 days. The ICPI is the mean score per bird per observation over the 8-day period. The most virulent viruses will give indices that approach the maximum score of 2.0. The ICPI of isolate RS-1177 was 0.0, therefore, not virulent.

The Mean death time (MDT) in eggs of isolate RS-1177 was also determined to check the pathogenicity. Briefly, five 10-day-old embryonating chicken eggs were infected with serial 10-fold dilutions of viruses (4HAU diluted 10^−2^ to 10^−5^). The eggs were incubated at 37°C and monitored twice daily for seven days. The time required to kill the embryos was recorded. The highest dilution that killed all embryos was considered as the minimum lethal dose. MDT was calculated as the mean time in hours required for the minimum lethal dose to kill the embryos and has been used to classify NDV strains into the following groups: velogenic (taking under 60 h to kill); mesogenic (taking between 60 and 90 h to kill); and lentogenic (taking more than 90 h to kill) [17]. The MDT of isolate RS-1177, like the positive control (APMV-1 vaccine strain), were more than 90 h (>148 h for the RS-1177 and >108 h for La Sota NDV strain), showing that this isolate indeed is a low pathogenic strain and in accordance with the molecular and ICPI results.

## Discussion

Although the RS-1177 sampled contained a coinfection of influenza A virus and paramyxovirus, it was obtained from an apparently healthy bird (*Calidris fuscicollis*) without clinical signs or apparent symptoms. This observation was consistent with the *in vivo* results and the absence of pathogenicity genetic signatures in the influenza A hemagglutinin cleavage site and in the cleavage site of the APMV F protein. The white-rumped sandpiper is a Nearctic long distance migrant that breeds in the northern tundra of Canada and Alaska and which spends much of its non-breeding period in southeastern South America, predominantly on the Patagonian coast [19]. The APMV isolated from RS-1177 had greatest similarity to a strain already found on the Brazilian coast [20] and first described in rockhopper penguins (*Eudyptes chrysocome)* from Falkland Islands, an archipelago in the South Atlantic Ocean on the Patagonian Shelf. White-rumped sandpipers are regularly spotted on Fracasso Beach, Argentina between February and April, with maximum numbers in March [21]. As we sampled the bird in April, we consider it most likely that we sampled it as it was returning to North America.

Next-generation sequencing, a metagenomics tool, is revolutionising the field of wildlife pathogen discovery by enabling the discovery of aetiological agents for which little or no prior information is available [22]. In our study we employed *de novo* assembly to identify a novel virus with high similarity to the *Avulavirus* genus. The genetic dissimilarity of this virus and the lack of cross-reactivity to other identified *Avulavirus* strongly suggests that it could be part of a new Avian Paramyxovirus group.

Further studies are needed to assess the host specificity, prevalence and pathogenicity of the virus. The detection of a new isolate, RS-1177, using the primer set described by Tong et.al, 2008 confirms the ability of this assay to detect novel paramyxoviruses in outbreaks and diseases of unknown etiology [13].

## Supporting information

S1 Table Estimates of evolutionary divergence between *Avulavirus* nucleotide sequences, including strain RS-1177. The number of base differences per site from between sequences are shown. All positions containing gaps and missing data were eliminated. Evolutionary analyses were conducted in MEGA6 [17].
(XLS)

S2 Table Antigenic analysis of RS-1177 by HI tests with standard antisera against representative APMV and influenza A virus strains. HI titer represents the reciprocal of the serum dilution inhibiting the activity of 4 hemagglutinating units of RS-1177.
(XLSX)

## References

[1] Briand FX, Henry A, Massin P, Jestin V. Complete genomes sequence of a novel avian paramyxovirus. J Virol. 2012; 86:7710 10.1128/JVI.00946-12 22733876 PMC3416274

[2] Terregino C, Aldous EW, Heidari A, Fuller CM, De Nardi R, Manvell RJ, et al Antigenic and genetic analyses of isolate APMV/wigeon/Italy/3920–1/2005 indicate that it represents a new avian paramyxovirus (APMV-12). Arch. Virol. 2013; 158: 2233–2243. 10.1007/s00705-013-1735-2 23708253

[3] ICTV—International Committee on Taxonomy of Víruses. Available from: http://www.ictvonline.org/vírusTaxonomy.asp. [2016 Oct 03].

[4] Miller PJ, Afonso CL, Spackman E, Scott MA, Pedersen JC, Senne DA, et al Evidence for a new avian paramyxovirus serotype 10 detected in rockhopper penguins from the Falkland Islands. J. Virol. 2010; 84: 11496–11504. 10.1128/JVI.00822-10 20702635 PMC2953191

[5] Karamendin K, Kydyrmanov A, Seidalina A, Asanova S, Sayatov M, Kasymbekov E, Khan E, Daulbayeva K, Harrison SM, Carr IM, Goodman SJ, Zhumatov K. 2016 Complete genome sequence of a novel avian paramyxovirus (APMV-13) isolated from a wild bird in Kazakhstan. Genome Announc 4(3):e00167–16. 10.1128/genomeA.00167-16 27198008 PMC4888989

[6] Goraichuk I, Sharma P, Stegniy B, Muzyka D, Pantin-Jackwood MJ, Gerilovych A, Solodiankin O, Bolotin V, Miller PJ, Dimitrov KM, Afonso CL. 2016 Complete genome sequence of an avian paramyxovirus representative of putative new serotype 13. Genome Announc 4(4):e00729–16. 10.1128/genomeA.00729-16 27469958 PMC4966462

[7] Thampaisarna R, Buic VN, Trinh DQ, Nagai M, Mizutani T, Omatsu T, Katayama Y, Gronsang D, Le DHT, Ogawa H, Imai K. Characterization of avian paramyxovirus serotype 14, a novelserotype, isolated from a duck fecal sample in Japan. Virus Res. 2017; 228: 46–57. 10.1016/j.virusres.2016.11.018 27884627

[8] Yamamoto E, Ito H, Tomioka Y, Ito T. Characterization of novel avian paramyxovirus strain APMV/Shimane67 isolated from migratory wild geese in Japan. J. Vet. Med. Sci. 2015; 77(9): 1079–1085. 10.1292/jvms.14-0529 25866408 PMC4591148

[9] Thomazelli LM, Araujo J, Ferreira CS, Hurtado R, Oliveira DB, Ometto T, et al Molecular surveillance of the Newcastle disease virus in domestic and wild birds on the North Eastern Coast and Amazon biome of Brazil. Rev Bras Cienc Avic. 2012; 14: 1–7.

[10] Araujo J, Azevedo SM Jr, Gaidet N, Hurtado RF, Walker D, Thomazelli LM, et al Avian Influenza Virus (H11N9) in migratory shorebirds wintering in the Amazon region, Brazil. PLoS ONE. 2014; 9(10):e110141 10.1371/journal.pone.0110141 25329399 PMC4199675

[11] Djikeng A, Halpin R, Kuzmickas R, Depasse J, Feldblyum J, Sengamalay N, et al Viral genome sequencing by random priming methods. BMC Genomics. 2008; 9: 5 10.1186/1471-2164-9-5 18179705 PMC2254600

[12] Rosseel T, Lambrecht B, Vandenbussche F, Vanden Berg T, Van Borm S. Identification and complete genome sequencing of paramyxoviruses in mallard ducks (Anas platyrhynchos) using random access amplification and next generation sequencing technologies. Virol J. 2011; 8: 463 10.1186/1743-422X-8-463 21978491 PMC3219605

[13] Tong S, Chern SW, Li Y, Pallansch MA, Anderson LJ. Sensitive and broadly reactive reverse transcription-PCR assays to detect novel paramyxoviruses. J Clin Microbiol. 2008 8; 46(8):2652–8. 10.1128/JCM.00192-08 18579717 PMC2519498

[14] Kolakofsky D, Roux L, Garcin D, Ruigrok RW. Paramyxovirus mRNA editing, the "rule of six" and error catastrophe: a hypothesis. J Gen Virol. 2005;86(Pt 7):1869–77. 10.1099/vir.0.80986-0 15958664

[15] Hall TA. 1999 BioEdit: a user-friendly biological sequence alignment editor and analysis program for Windows 95/98/NT. Nucl. Acids. Symp. Ser. 41:95–98

[16] Tamura K. and Nei M. Estimation of the number of nucleotide substitutions in the control region of mitochondrial DNA in humans and chimpanzees. Molecular Biology and Evolution. 1993 10:512–526. 8336541 10.1093/oxfordjournals.molbev.a040023

[17] Tamura K, Stecher G, Peterson D, Filipski A, and Kumar S (2013). MEGA6: Molecular Evolutionary Genetics Analysis version 6.0. Molecular Biology and Evolution 30: 2725–2729. 10.1093/molbev/mst197 24132122 PMC3840312

[18] OIE (Office International des Epizooties) Newcastle disease, 2004. OIE Manual of Standards for Diagnostic Tests and Vaccines, chapter 2.1.15.

[19] Hayman P, Marchant J, Prater T. Shorebirds: An identification guide. Boston: Houghton Mifflin 1986: 412 p.

[20] Fornells LAMG, Silva TF, Bianchi I, Travassos CEPF, Liberal MHT, Andrade DM, et al Detection of paramyxoviruses in Magellanic penguins (Spheniscus magellanicus) on the Brazilian tropical coast. Vet Microbiol. 2012;156:429–433. 10.1016/j.vetmic.2011.11.026 22189432

[21] Costa ES, Ayala L, Sul JAI, Coria NR, Sánchez-Scaglioni RE, Alves MAS, et al ANTARCTIC AND SUB-ANTARCTIC SEABIRDS IN SOUTH AMERICA: A REVIEW. Oecol. Aust. 2011; 15(1): 59–68.

[22] White DJ, Hall RJ, Wang J. Virus Genes. 2016; 52: 727 10.1007/s11262-016-1342-x 27115421

